# Algorithmic indexing in MEDLINE frequently overlooks important concepts and may compromise literature search results

**DOI:** 10.5195/jmla.2025.1936

**Published:** 2025-01-14

**Authors:** Alexandre Amar-Zifkin, Taline Ekmekjian, Virginie Paquet, Tara Landry

**Affiliations:** 1 alexandre.amar-zifkin@umontreal.ca, Bibliothèque de la Santé, Université de Montréal, Montréal, Québec, Canada; 2 taline.ekmekjian@phac-aspc.gc.ca, Public Health Agency of Canada library, Office of the Chief Science Officer, Public Health Agency of Canada, Ottawa, Ontario, Canada; 3 virginie.paquet@umontreal.ca, Bibliothèques de l'Université de Montréal, Montréal, Québec, Canada; 4 tara.landry@umontreal.ca, Bibliothèque de la Santé, Université de Montréal, Montréal, Québec, Canada

**Keywords:** Abstracting and Indexing, Algorithms, Medical Subject Headings, PubMed, MEDLINE, Search Strategies, Database Searches, Information Storage, Retrieval, MeSH

## Abstract

**Objective:**

To evaluate the appropriateness of indexing of algorithmically-indexed MEDLINE records.

**Methods:**

We assessed the conceptual appropriateness of Medical Subject Headings (MeSH) used to index a sample of MEDLINE records from February and March 2023. Indexing was performed by the Medical Text Indexer-Auto (MTIA) algorithm. The primary outcome measure is the number of records for which the MTIA algorithm assigned subject headings that represented the main concepts of the publication.

**Results:**

Fifty-three percent of screened records had indexing that represented the main concepts discussed in the article; 47% had inadequacies in the indexing which could impact their retrieval. Three main issues with algorithmically-indexed records were identified: 1) inappropriate MeSH assigned due to acronyms, evocative language, exclusions of populations, or related records; 2) concepts represented by more general MeSH while a more precise MeSH is available; and 3) a significant concept not represented in the indexing at all. We also noted records with inappropriate combinations of headings and subheadings, even when the headings and subheadings on their own were appropriate.

**Conclusions:**

The indexing performed by the February-March 2023 calibration of the MTIA algorithm, as well as older calibrations, frequently applied irrelevant or imprecise terms to publications while neglecting to apply relevant terms. As a consequence, relevant publications may be omitted from search results and irrelevant ones may be retrieved. Evaluations and revisions of indexing algorithms should strive to ensure that relevant, accurate and precise MeSH terms are applied to MEDLINE records.

## INTRODUCTION

The purpose of indexing, or “the act of describing or identifying a document in terms of its subject content” [1], is to make pertinent documents retrievable. Controlled vocabulary terms provide indexers with specific, preferred entries for concepts that can manifest in multiple, synonymous ways, and have been deployed in many different bibliographic databases and research domains [2]. The Medical Subject Headings (MeSH) thesaurus, developed by the United States National Library of Medicine (NLM) in 1960, is the controlled vocabulary of terms “used for indexing, cataloging, and searching for biomedical and health-related information and documents” [3], and is used in the NLM catalog and in MEDLINE, the premier bibliographic database in biomedical sciences.

For decades, countless health and information professionals have taught and been taught that using MeSH in their literature searches in MEDLINE will increase the precision of their queries, thus improving the relevance of their results [4, 5]. A classic demonstration of the usefulness of MeSH is that a study excluding patients with diabetes would not receive a MeSH term indicating diabetes, whereas a text word search for ‘diabetes’ would irrelevantly retrieve that publication if it had mentioned its exclusion criteria in the title or abstract. Searchers are taught that MeSH terms assigned to a record reflect the main concepts of an article and thus reliably indicate its most important aspects [6–8]. As evidence of the presumed value and utility of controlled vocabulary such as MeSH to indicate aboutness, resources like the Cochrane Handbook, which also influence searching practices more broadly, direct searchers to use them [9].

Indexing of biomedical literature has been moving gradually from manual or human-based indexing towards automatic semantic indexing for more than a decade [10]. The movement to automation aims to reduce time-to-indexing and cost, identified in Mao and Lu [11] as 2–3 months and $10 per publication, respectively, and must be considered in the context of the ever-increasing volume of biomedical literature. For example, 1,279,327 indexed citations were added to MEDLINE in 2023, compared to 734,052 in 2013, a 74% increase over 10 years [12].

In December 2021, the NLM announced its intention that “all citations indexed for Medline will be indexed by MTIA” (Medical Text Indexer-Auto) [13]. Although algorithmic indexing has provided indexing suggestions to human indexers since 2002 via the Medical Text Indexer (MTI) [14], the move towards fully automated indexing significantly changes how bibliographic records are indexed [15]. Humans with subject matter expertise previously determined central elements of articles and selected appropriate MeSH terms for them; as of April 2022, the front line of indexing for all records is performed by an algorithm, with humans limiting their curation to sets involving genes and proteins [13].

Briefly, the MTIA algorithm determined which MeSH terms to apply to a record by:

- identifying uncommon or specific terms in that article's title and abstract;
- finding MeSH for those terms;
- gathering MeSH which have been assigned to other records with similar uncommon or specific terms from within MEDLINE; and
- ranking these terms and deciding which to apply to the record [16].
  - The MTIA used several processes to rank terms. One such step involved the prioritization of a subheading over a MeSH heading when a term was present in both thesauri (e.g.: the subheading *pharmacokinetics* would be preferred over the MeSH term *Pharmacokinetics*). (*See Section 13. MH/SH Substitution*) [16].

Unlike human indexers, MTIA did not consider the journal in which an article appeared, the author-suggested keywords, or the full text of the article, any of which might provide more insight into concepts germane to the aboutness of the article. For example, studies have found that the methods section can also be quite informative, often containing information on species, sex, and age groups, all of which are required by MeSH indexing guidelines as check tags [17, 18].

Previous research has identified issues with indexing in MEDLINE. Two such studies, Minguet et al [19] and Tonin et al [20], found that indexing in pharmacy publications did not consistently use MeSH terms from the Pharmacy branches of the MeSH tree, while Portaluppi [21] arrived at a similar conclusion for articles on chronobiology. Layton and Clarke [22] found that the representation of statistical concepts in interventional dentistry publications was lacking, and Wilczynski and Haynes [23] identified issued with the consistency and accuracy of indexing of knowledge syntheses. Moore, Yaqub and Sampat [24] explored the classification of documents by disease area and Neveol et al [8] and Rae et al [25] identified challenges in pairing main headings and subheadings.

A more recent study sought to assess the outputs of algorithmic indexing by taking a sample of records published in the year 2000, before human indexers began to receive indexing recommendations from the MTI, inputting those records into a public-facing MTI, and comparing the MTI indexing to that of humans [26]. While this identified some issues with MTI outputs, notably in headings representing populations (‘check tags’) and the influence of sentence structure on concept ranking, it is ultimately a comparison of algorithmic indexing output to human indexing, and only lightly questions the appropriateness of the terms assigned by the algorithm. By contrast, our study seeks to determine whether automatically assigned index terms reflect the main concepts of an article and indicate its most important aspects.

In the months preceding this research, the authors each encountered multiple MEDLINE records where the indexing (later determined to be automated) did not align with their experience or expectations. For example, for the article, *An exploratory study on support for caregivers of people with vision impairment in the UK* [27] there is no indexing representing visually impaired persons, or even of visual impairment. By contrast, *Laparoscopic versus open elective right hemicolectomy with curative intent for colon adenocarcinoma* [28] is indexed with the MeSH term *Child, Preschool* for no apparent reason, with no other MeSH indicating the correct age range of study participants.

The first rule of indexing is to include all topics known to be of interest to users that are treated substantively within the document [29]. Although some indexing theorists argue that there can be no single “correct” set of index terms for a document, as different requesters may seek out the same document for different reasons [30], we would argue that, given the purpose of indexing – to make documents retrievable – it is reasonable to assert that the *essential* topics, or concepts, of a document should be accurately represented in its indexing.

These most recent steps towards fully automated and algorithmic indexing, even with human spot-checks, raise fundamental questions:

- How well do algorithmically-applied MeSH indicate a publication's essential concepts?
- When information specialists and health professionals run MeSH-only searches, expecting that MeSH will identify relevant research on their topics, is the algorithm causing anything to be overlooked? If so, what?

If algorithmically applied MeSH are found to frequently overlook or misrepresent salient concepts of publications, how much confidence should instructors of literature searching to tomorrow's health professionals have in their teaching of MeSH as a first step to quickly and precisely identifying the best available evidence?

We recognize that as of late April 2024, MTIA has been replaced by MTIX [Medical Text Indexer-NeXt Generation], which uses a machine-learning algorithm rather than a rules-based algorithm [31]. Nonetheless, as searchers and researchers continue to engage with recent, and therefore algorithmically-indexed, publications, we hope that the following analyses and insights into automated indexing lay a foundation for a deeper and broader understanding of indexing algorithms and their outputs.

## METHODS

This study assessed whether a sample of MTIA-indexed articles were indexed with MeSH that adequately described their main concepts. As such, this research should not be interpreted as comparing algorithmically-indexed records to human-indexed records.

We piloted our screening process with a set of 10 records drawn from January 2022 and 2023. Our team of librarians, each with significant expertise in database searching and instruction, independently assessed each record and determined whether the assigned MeSH terms adequately represented main concepts. We found that our team was generally able to identify similar main concepts for each article within the set, and likewise determine whether the assigned MeSH was concordant with those concepts. However, the team struggled to identify major concepts in articles that were not based in clinical health sciences, stemming instead from the broader constellations of life sciences such as genetics and zoology, as well as civil engineering, agricultural sciences and software development. We therefore decided to allow screeners to exclude these articles when screening our final set.

Our final sample was sourced using Ovid MEDLINE® ALL. On March 31, 2023, we sampled 20 days of MEDLINE between February 6 and March 7, 2023, skipping weekends and holidays so that each date had a similar number of publications. We opted for a recent date range because the algorithm can be recalibrated over time, and we wanted to assess recently indexed publications rather than records that might have been indexed using a previous configuration.

We only used records with “automated” in the Indexing Method [IG] field. This excluded records with no value in the IG field, indicating entirely human indexing, and records with a value of “curated”, indicating revision by human indexers of terms applied by MTIA. We used a random sequence generator [32] to select 50 records from each date, and used Ovid's internal deduplication function to remove duplicate records. Our final sample consisted of 998 unique records. Sample queries for date and randomization are presented below:

1. 20230206.ed and automated.ig and medline.st and english.la (3420)
2. from 1 keep [50 unique random numbers between 1-3420] (50)

We exported the 998 records into a spreadsheet. Each record was assigned to two screeners, who were blinded to each other's work and to the MeSH terms that had been assigned to each record by the MTIA. Because our final sample was 100x larger than our test set, we established a two-step screening protocol. The first pass of screening allowed for a record to be excluded if there was insufficient information in the record—for example, if there was no abstract and the title was uninformative – or if the screener considered the article to be outside the scope of clinical health sciences.

An example of a record both without an abstract and with an uninformative title is the article *Blue as an Orange* [33]. An example of a record that was considered to be outside the scope of clinical health sciences is *Mathematical analysis of topological and random m-order spread models* [34].

In the second pass of screening, each screener assigned concepts based on the available data in the record, either using MeSH headings of which they were already aware (e.g.: Patient Education As Topic) or more general terminology (e.g.: air quality or air pollution). Screeners were not limited in the number of concepts they could assign, so long as they felt the concepts were descriptive of an aspect of the publication. As Publication Types are not conceptual, per se, we did not direct screeners to apply publication types like ‘Randomized Controlled Trial’, but we did encourage the use of ‘As Topic’ terms when appropriate (e.g. ‘Randomized Controlled Trial As Topic’). Screeners were instructed to consider the article they were screening as a ‘seed’ or ‘target’ article, and assign concepts that they felt would be part of a search for the article, as well as articles on a similar topic, within the database.

Once a screener had identified the main concepts of a record, they were un-blinded to the MeSH assigned to the article. The screener would then indicate agreement or disagreement between their concepts and the MeSH assigned by MTIA. In cases of uncertainty or ambiguity, MeSH scope notes were consulted as needed to ensure understanding of a term was accurate. Three abridged rows from the screening tool, showing assessments from one screener, are provided in Table 1. The “yes” in the Agreement? column indicates that the concepts identified by the screener were deemed present in the indexing. Our complete dataset is available online at https://osf.io/ckj3m/.

**Table 1:** Sample records in screening form

Record	Screener-identified concepts	MeSH assigned by MTIA	Agreement?
Indications for continuous electroencephalographic (cEEG) monitoring: What do they tell us? *Epilepsy Research* [35]	EEG Epilepsy Length of monitoring?	Female Humans Middle Aged Male Prospective Studies Epilepsy/di [Diagnosis] ^*^Epilepsy Monitoring, Physiologic Electroencephalography ^*^Lupus Erythematosus, Systemic	Yes
Development and validation of a nomogram for evaluating the incident risk of carotid atherosclerosis in patients with type 2 diabetes. *Frontiers in Endocrinology* [36]	Carotid atherosclerotic disease Type 2 diabetes Risk	Humans ^*^Diabetes Mellitus, Type 2 Nomograms ^*^Non-alcoholic Fatty Liver Disease ^*^Carotid Artery Diseases Risk Factors	Yes
The trends and determinants of seasonal influenza vaccination after cardiovascular events in Canada: a repeated, pan-Canadian, cross-sectional study. *Health Promotion and Chronic Disease Prevention in Canada* [37]	Canada flu vaccination/vaccination cardiovascular diseases public policy	Humans Canada/ep [Epidemiology] Cross-Sectional Studies Influenza, Human/ep [Epidemiology] Influenza, Human/pc [Prevention & Control] ^*^Influenza, Human Seasons Vaccination	No (missing cardiovascular diseases)

Disagreements between screeners–where one screener indicated that their concepts and the MeSH aligned, while those of the other screener did not–were resolved through discussion by two authors (AA-Z and TL), who were blinded to the identities of the disagreeing screeners. The results of the analysis are presented below.

## RESULTS

From our sample of 1,000 records, 2 duplicates were removed.

From the 998 records which were screened, we excluded 287 because they did not contain enough information to assign concepts or because they were outside what we considered as the scope of health sciences. The remaining 711 records were screened by our team. The flow of record screening is presented in the PRISMA-styled figure below.

**Figure 1:**
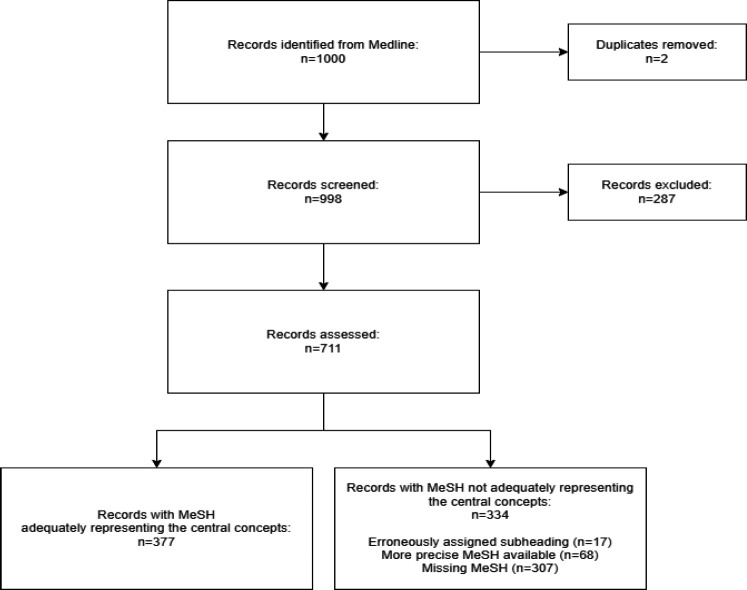


After resolving disagreements, we found that 377 records (53%) had been assigned MeSH terms that adequately represented the main concepts present in the title and abstract and 334 records (47%) had one or more deficiencies in their indexing. The team found that these 334 records had commonalities which we have grouped into four main categories, with varying potential impacts on retrieval. These are summarized in Table 2.

**Table 2:** Results

Indexing issues	Number of records^*^	% of records assessed	Example
Appropriate subheadings erroneously assigned to a main heading	17	2.4%	Safety and tolerability of obeticholic acid in chronic liver disease: a pooled analysis of 1878 individuals [38]. The subheading of drug therapy is attached to tire MeSH for pruritis, tire adverse effect of obeticholic acid discussed in the abstract. This linkage implies that the subject of the article is drug therapy for the adverse effect rather than drug therapy for a condition (Non-alcoholic Fatty Liver Disease/dt [Drug Therapy]), an adverse effect of the drug (Chenodeoxycholic Acid/ae [Adverse Effects]), and a chemically-induced adverse effect (Pruritis/ci [Chemically Induced])
Concept represented by MeSH while a more precise MeSH was available	68	9.6%	Ambulatory oxygen therapy in lung transplantation candidates with idiopathic pulmonary fibrosis referred for pulmonary rehabilitation [39] Indexed with Oxygen, without tire therapeutic use subheading and without the more precise Oxygen Inhalation Therapy heading.
Significant concept not represented in tire indexing at all	307	43.2%	Bridging knowledge gaps in paediatric chronic urticaria through a video-based educational tool [40] There is no representation of anything relating to education in the indexing, despite Patient Education as Topic being available.
MeSH terms adequately represented the main concepts present in the title and abstract	377	53.0%	Development and Validation of the HIV-CARDIO-PREDICT Score to Estimate the Risk of Cardiovascular Events in HIV-Infected Patients [41] HIV infections, Cardiovascular Diseases, and Risk were all represented in the indexing.

* While 334 records had indexing issues, some had more than one.

## DISCUSSION

This study identified a number of indexing issues for publications that were indexed using MTIA. Three of the issues we identify may result in work arounds that could significantly and undesirably increase the number of retrieved citations. For instance, researchers conducting knowledge syntheses could bypass the issue of appropriate subheadings being erroneously assigned to a main heading by using ‘floating’ subheadings in their searches (to retrieve any record indexed with a specific subheading, regardless of which heading it is attached to).

For the 9.6% of records identified, where records were represented by broader MeSH while more precise MeSH terms were available, searchers would need to include these broader terms in their search queries in order to retrieve these publications. For example, when searching for articles about the experiences of visually impaired persons, rather than simply searching for Visually Impaired Persons, a searcher now needs to include less precise terms from the Vision Disorders and Eye Diseases MeSH trees, potentially going further to Eye, Optometry, Ophthalmology, Optometrists or Ophthalmologists. Although health educators, information professionals [6] and the NLM itself [42] have explicitly or tacitly recommended searching by the most precise MeSH, this may result in the exclusion of articles which have been indexed using MTIA. In practical terms, this means that, because the algorithm cannot “read between the lines” searchers must replace a precise MeSH heading with MeSH terms tangentially related to their topic.

For records in which a significant concept was not represented in the indexing, the impact on searching is clear. Because they were not assigned appropriate headings for a central concept, a MeSH only search, no matter how expansive, will not retrieve those records because they were not assigned appropriate headings for a central concept. Guo, Gotz and Wang [43] conceptualize the omission of a relevant concept from an article's indexing as a bottleneck in the search. As a consequence, a searcher would only find relevant publications through title-abstract-keyword searching, which may introduce noise into search results.

In the course of our screening, our team founds records where the MTIA assigned somewhat florid and unusual indexing terms that did not pertain to the concepts present in the title or abstract. As these findings were incidental and would likely not result in the exclusion of relevant publications from a search, we did not systematically note them. Nevertheless, we felt it was important to discuss them here, as other health information professionals may have encountered similar instances in their own search results.

For example, we found several instances of MTIA erroneously assigning a MeSH term based on the use of an acronym or evocative language in the title or abstract. For instance, the article *Bridging knowledge gaps in paediatric chronic urticaria through a video-based educational tool* [40] was indexed with the MeSH term Copper, likely because the authors abbreviated ‘chronic urticaria’ as CU (the chemical symbol for copper). Likewise, we found that the use of metaphor, simile or rhetoric to describe or illustrate ideas sometimes led to indexing errors, such as the article *Not all cauliflowers are HPV: challenge* [44] being assigned the MeSH term Brassica, despite being about cauliflower-shaped genital warts and not the noble brassica family.

We also found several instances of irrelevant indexing as a result of the MTIA assigning MeSH terms based on the indexing of similar records within the database (“neighboring records”) [16]. This type of error is illustrated by the indexing of *Prevalence of undernutrition and its associated factors among older adults using Mini Nutritional Assessment tool in Womberma district, West Gojjam Zone, Amhara Region, North West Ethiopia, 2020* [45]. Although about undernutrition among older adults in Ethiopia, the record was indexed with Infant, Newborn, likely because more records in MEDLINE about undernutrition in Ethiopia are concerned with infants (275 records) than older adults (26 records, both as of November 2023). Although records with these types of errors are relatively quick to exclude from search results, it is important to note that this type of indexing issue will become more frequent if algorithmically-indexed records form the foundation of future algorithmic indexing [46].

Finally, we found several examples of the MTIA improperly indexing a publication with the population being excluded, as illustrated by the article *Impact of the Controlling Nutritional Status (CONUT) score as a prognostic factor for all-cause mortality in older patients without cancer receiving home medical care: hospital ward-based observational cohort study* [47]. Despite expressly being about patients without cancer, the MTIA assigned the MeSH terms Neoplasms/Therapy, as well as Neoplasms as a major topic to its indexing.

## LIMITATIONS AND FUTURE DIRECTIONS

Our study has significant limitations. First, we are not trained indexers. Our research did not seek to assess MTIA adherence to indexing guidelines, but rather sought to assess whether terms assigned by MTIA reflect the main concepts of an article and indicate its most important aspects. Consequently, our assessments of the representation of certain concepts may not have fully aligned with NLM indexing guidelines [48].

Another limitation is that publications from outside of our areas of experience were excluded. As medical librarians, our familiarity with MeSH is the result of years of literature searches supporting clinicians and knowledge synthesis projects [49–51] and providing instruction to health professionals. We found that we struggled to identify concepts from the titles and abstracts of records stemming from outside of clinical health; a difficulty also encountered in Liu and Wacholder [52]. We chose to exclude these articles because we were concerned that our inability to determine which parts of these publications would be relevant for a searcher would introduce inconsistency into our appraisal of MTIA performance.

We also note that we did not review screeners' individual assessments of the essential concepts within a record, nor did we check to make sure screeners agreed as to what those concepts were. Given the subjective nature of assigning subjects to a document, Golub's *A framework for evaluating automatic indexing or classification in the context of retrieval* [30] recommends that evaluations of indexing quality begin with expert consensus on all the relevant, appropriate subjects that should be assigned to it. Therefore, we recognize that our assessment of MTIA performance would be more solid if built on that additional foundation.

As previously noted, as of late April 2024, MTIA has been discontinued in favour of MTIX. In the March-April 2024 issue of its technical bulletin, the NLM highlights that the training data for MTIX is more recent, dating from 2007 through 2022, that MTIX considers the journal title where MTIA did not, and that MTIX can recognize the concept of “Hip Fractures” from interrupted and reordered phrases like “complex fractures and dislocations of the hip” [31]. The same publication asserts that the MTIX performance is comparable to that of MTIA at the level of precision (not erroneously applying terms) while improving on MTIA's recall (applying a greater number of terms which are not incorrect). However, MTIX, like MTIA, still does not access the full text of the publications it indexes. Moving forward, an obvious future direction would be the replication of this research with records indexed by MTIX.

We concur with Chen, Bullard and Giustini [26] that future research should ensure samples from a wide breadth of publications to assess the quality of indexing algorithm outputs in different fields. For example, Moore, Yaqub and Sampat [24] found that MTIA performed well for subject areas with specific terminology, such as diseases, and the NLM has indicated that chemicals and genes are priorities [53]. However, ensuring equitable quality of indexing across subjects will require ongoing research to evaluate indexing algorithm outputs in areas with innate lexical ambiguities like nursing, education or continuity of patient care.

We posit that future research should particularly scrutinize the accuracy of indexing for populations and its effects on retrieval. Some articles only identify populations of interest in their full text. As an example, Buono et al [54] has no indication of Black or African American people in the title or abstract, but on the basis of its full text, it has been indexed with the MeSH ‘Black or African American’. As no MEDLINE platform presently permits a user to search within full text, MeSH indicating population groups, applied by human indexers based on the full text, constitute the only means for a searcher to find articles relevant to those groups. Further research could also appraise any disproportionate changes in numbers of records receiving MeSH for specific population subgroups or concepts.

Finally, when central concepts such as species, populations or publication types are omitted or inaccurately represented in indexing, search strategies or filters relying on MeSH-only queries may inadvertently overlook or exclude relevant publications. Filters designed and validated in a time when indexing was performed by humans, including such touchstones as the Cochrane Highly Sensitive Search Strategies [55], which has a MeSH-only line to exclude non-human animals, should be re-evaluated, as their performance may no longer be as reliable in this brave new world of inhuman indexing.

## Data Availability

Data associated with this article are available in the Open Science Framework at https://osf.io/ckj3m/.

## NOTE

Preliminary findings were presented the 2023 Canadian Health Library Association - Association des bibliothèques de la santé du Canada (CHLA-ABSC) conference; the authors also contributed commentary to the *Journal of European Association for Health Information and Libraries (JEAHIL)*.
